# Self-confidence in oral and maxillofacial surgery: a cross-sectional study of undergraduate dental students at Kuwait University

**DOI:** 10.1186/s12909-021-02614-x

**Published:** 2021-04-07

**Authors:** Mohammad Kamal, Mohammad Abdulwahab

**Affiliations:** Department of Surgical Sciences, Faculty of Dentistry, Kuwait University, Health Sciences Centre, Safat, Kuwait

**Keywords:** Dental students, Self-confidence, Oral surgery education, Oral and maxillofacial surgery teaching

## Abstract

**Background:**

To evaluate the self-confidence of undergraduate dental students in relation to oral and maxillofacial surgery (OMFS) to assess the teaching curriculum at Kuwait University using a validated questionnaire originally developed by the Association of British Academic Oral Maxillofacial Surgeons (ABAOMS).

**Methods:**

A cross-sectional survey of sixth year (*n* = 20) and seventh year (*n* = 19) dentistry students was conducted by Kuwait University Faculty of Dentistry between the 1st and 15th of May 2020. The ABAOMS questionnaire is composed of 17 questions assessing various areas of the undergraduate OMFS curriculum. The response options to the questionnaire utilised a Likert scale. Independent sample t-tests were performed to assess the difference in responses between the 2 year groups. Spearman’s rho correlations were calculated to measure the strength of association between confidence in all aspects of surgical and forceps exodontia.

**Results:**

A total of 39 questionnaires were completed by the students. The majority of students expressed feelings of confidence that they have enough knowledge to undertake independent practice (61%). General aspects of the questionnaire were answered favourably except for surgical extraction of teeth, in which both classes reported a lower level of self-confidence.

**Conclusions:**

The ABAOMS survey revealed the students’ self-confidence in undertaking independent practice and preforming basic oral surgery procedures. Students felt comfortable with exodontia using forceps and elevators, root removal, managing acute pericoronitis, managing haemorrhage from a socket, assessing impacted teeth, and recognising the clinical features of potentially malignant and malignant lesions of the oral cavity. They reported a lower level of confidence in performing surgical procedures.

## Background

A dental school teaching curriculum aims to provide students with the highest theoretical and practical education to prepare for clinical practice. Several educational schemes and curricula have been developed across academic institutions internationally to enhance undergraduate education in Oral and Maxillofacial Surgery (OMFS) [1–3]. A stepwise approach to teaching practical procedures and thorough clinical assessments has allowed instructors to assess the students’ educational level and prepare them for the challenges of surgery [1, 3–5]. Consequently, to enhance the teaching experience and constant refinement of the OMFS teaching curriculum and clinical competency assessments, survey instruments have been developed to evaluate the self-confidence of undergraduate dental students in performing OMFS procedures. Moreover, these survey instruments are also essential to enhance educational quality and teaching effectiveness based on their feedback [3, 4, 6, 7]. The Association of British Academic Oral Maxillofacial Surgeons (ABAOMS) educational committee has designed a validated survey instrument to assess dental students’ self-confidence in OMFS and has been used in several countries [7–10]. The ABAOMS instrument is made of 17 questions assessing the student’s confidence in performing oral surgery procedures, the role of outreach in oral surgery, anatomy teaching concerning oral surgery, and oral surgery career aspiration [7].

The Faculty of Dentistry at Kuwait University began the undergraduate OMFS teaching curriculum in 2002 for dental students during their clinical training years. Kuwait University follows a 7-year dentistry programme, in which the students start their clinical training in the fifth year of the programme. Courses focusing on OMFS are taught during the fifth, sixth and seventh years of training. Students’ perceptions of the OMFS training they receive have received little attention. There have been no prior studies assessing students’ views on their oral surgery education and their confidence in conducting surgical procedures.

The aim of this study was to evaluate the dental students’ perceptions of the undergraduate OMFS teaching curriculum using the ABAOMS questionnaire and assess their self-confidence and readiness to perform common oral surgical procedures.

## Methods

We conducted a cross-sectional survey of all 6th and 7th-year dentistry students in the Kuwait University Faculty of Dentistry between 1 May and 15 May 2020.

A previously validated questionnaire by ABAOMS was used as the survey instrument the study [7, 10]. The survey was distributed electronically using Google Forms (https://www.google.com/forms) to all eligible students at Kuwait University via the student portal network. Students consented to fill out the survey instruments voluntarily, and the responses remained anonymous throughout the study. Students provided information about age, gender, year of study, and the number of extractions performed, along with 17 questions assessing various areas of the undergraduate OMFS curriculum. The domains included minor oral surgery procedures usually performed by practicing dentists, such as extracting teeth and retained visible roots, and additional surgical management of a failed extraction such as raising of a mucoperiosteal flap, bone removal, root sectioning, and wound suturing. Students were also asked about their confidence levels in diagnosing and managing acute pericoronitis, management of post-operative haemorrhage from a socket, assessing impacted third molars with respect to guidelines, and recognising the clinical features of potentially malignant and malignant lesions of the oral cavity. Responses to the questions were in the form of a Likert scale, with five options ranging from strongly agree (1) to strongly disagree (5). All questions were answered completely by each participant.

A pilot survey was performed on four randomly selected students (two from their sixth year and two from their seventh year) to assess the questionnaire, especially for comprehension of the English language, and clarity of the question. The questionnaire was then also assessed by two faculty members, other that the authors, to check for consistency, clarity of the language, and understanding the aim of the questions. All feedbacks were constructive, and the authors felt comfortable to proceed with the survey instrument in its current form and structure. The students were informed verbally during an OMFS didactic session about the importance of self-reported confidence and outcomes to us as a faculty to further the curriculum and teaching goals. The students were reassured that all answers remained anonymous, and this was an evaluation of their confidence level to perform procedures. The questionnaire included an introductory cover letter that included the topic and objectives of the research, the procedure for answering the questions, the number of questions and duration needed to complete the questionnaire, and it included the following sentences: “There are no risks to you if you participate in this research. Your participation will increase knowledge about this important issue. All information collected will remain confidential. Neither your name nor address will be recorded in any assessment. There is no obligation for you to participate, and you have the freedom to agree or not agree to participate. This will not have any effect on your academic standing, your right to receive health care, or your employment status. You may withdraw from the research at any time. This research does not include any medical experiments, taking biological samples, or intervening any treatment plan set by your physician.” The students then had to indicate if they were willing to participate or not. All students present in the country have responded to the questionnaire. Only students who were not present in the country during the study did not participate in the study.

### Statistical analysis

The data was analysed using SPSS version 23 (SPSS; IBM Company, Chicago, IL, USA). Responses to the questionnaire were summarised as counts and percentages and compared according to the year of study (sixth versus seventh year). Independent sample t-tests were also performed to assess the difference in responses between the two groups by using the Likert responses as a continuous variable. Spearman’s rho correlations were calculated to measure the strength of association between confidence in all aspects of surgical and forceps exodontia. Additional analysis was performed to measure the Spearman’s rho correlations between confidence in surgical and forceps exodontia and anatomical teaching as well as the number of previous extractions performed. For all analyses, a *p*-value (two-tailed) of less than 0.05 was considered statistically significant.

### Ethical considerations

Students gave informed consent to participate in the survey voluntarily. All responses remained anonymous throughout the study. The study protocol was approved by the Ethical Committee at Kuwait University Health Sciences Centre, according to the Helsinki Declaration.

## Results

A total of 39 questionnaires were completed, representing a response rate of 90.9% for the sixth year students (*n* = 20), and 90.47% of the seventh year students (*n* = 19). Table 1 shows the demographic characteristics of the study participants and the number of extractions performed by respondents to date. Thirty-seven respondents were female (94.9%), and the mean age was 23.25 years for sixth year students and 24.26 years for seventh year students. The seventh year students had greater experience in tooth extractions with 78.9% of students having performed more than 25 extractions compared to 35% of the sixth year students.

**Table 1:** Students characteristics and number of extractions performed

Group	Respondents	Response rate	Age – (years)	Gender	Number of extractions performed – n (%)				
Year of Study	n	%	Mean	n (%)	< 10	10–15	15–20	20–25	> 25
6th year	20	90.90%	23.25	Male - 1 (5%) Female - 19 (95%)	3 (15%)	2 (10%)	4 (20%)	4 (20%)	7 (35%)
7th year	19	90.47%	24.26	Male - 2 (10.53%) Female - 17 (89.47%)	0	1 (5.3%)	0	3 (15.8%)	15 (78.9%)
Total	39	90.79%	23.76	Male - 3 (7.69%) Female - 36 (92.31%)	3 (7.7%)	3 (7.7%)	4 (10.3%)	7 (17.9%)	22 (56.4%)

The Likert responses to each question in the ABAOMS survey instrument regarding confidence in oral surgery procedures, dental anatomy and enjoyment of oral surgery are shown in Table 2. The majority of students expressed their confidence in having enough knowledge to undertake independent practice (61%). The difference in confidence between the seventh year students compared to sixth year students (78.9% vs 45%) was borderline significant (*p* = 0.051). Both sixth and seventh year students felt confident that they could extract an upper single-rooted tooth with an intact crown (94.7 and 90% respectively). Both groups were also confident that they could remove the visibly retained roots of an upper left first molar with elevators or forceps (84.2 and 65% respectively).

**Table 2:** The frequency of answers for each of the questions; % response by Likert scale (Independent t-test)

Question/Statement	Class	Strongly agree	Agree	Unsure	Disagree	Strongly Disagree	Mean ± STD	***p***-value
**B1.** The teaching that I have received in oral surgery has given me sufficient knowledge to undertake independent practice	6th year	2 (10%)	7 (35%)	7 (35%)	4 (20%)	0	2.65 (±0.93)	0.051
	7th year	5 (26.3%)	10 (52.6%)	2 (10.5%)	2 (10.5%)	0	2.05 (±0.91)	
**B2.** I feel confident that I could extract an upper single- rooted tooth with an intact crown, in an otherwise intact dentition	6th year	11 (55%)	9 (45%)	0	0	0	1.45 (±0.51)	0.161
	7th year	16 (84.2%)	2 (10.5%)	1 (5.3%)	0	0	1.21 (±0.54)	
**B3.** I feel confident that I could remove visible retained roots of an upper left first molar with elevators or forceps	6th year	6 (30%)	7 (35%)	3 (15%)	4 (20%)	0	2.25 (±1.1)	0.157
	7th year	11 (57.9%)	5 (26.3%)	0	3 (15.8%)	0	1.74 (±1.10)	
**B4.** I feel confident to assess and perform the surgical management of a failed extraction (e.g. a lower second molar) necessitating:								
**B4a.** The raising of a mucoperiosteal flap	6th year	0	2 (10%)	0	9 (45%)	9 (45%)	4.25 (±0.91)	< 0.001
	7th year	0	6 (31.6%)	8 (42.1%)	3 (15.8%)	2 (10.5%)	3.05 (±0.97)	
**B4b.** Bone removal	6th year	0	1 (5%)	5 (25%)	6 (30%)	8 (40%)	4.05 (±0.94)	0.002
	7th year	0	6 (31.6%)	8 (42.1%)	3 (15.8%)	2 (10.5%)	3.05 (0.97)	
**B4c.** Sectioning the tooth to facilitate elevation of the roots	6th year	0	1 (5%)	0	9 (45%)	10 (50%)	4.40 (±0.75)	< 0.001
	7th year	0	6 (31.6%)	9 (47.4%)	2 (10.5%)	2 (10.5%)	3.00 (0.94)	
**B4d.** Wound closure using appropriate suture materials	6th year	2 (10%)	7 (35%)	4 (20%)	3 (15%)	4 (20%)	3.00 (±1.34)	0.002
	7th year	11 (57.9%)	5 (26.3%)	2 (10.5%)	0	1 (5.3%)	1.68 (±1.06)	
**B5.** I feel confident to diagnose and manage acute pericoronitis	6th year	6 (30%)	10 (50%)	4 (20%)	0	0	1.90 (±0.72)	0.082
	7th year	13 (68.4%)	3 (15.8%)	3 (15.8%)	0	0	1.47 (±0.77)	
**B6.** I feel confident to manage haemorrhage from a socket	6th year	5 (25%)	9 (45%)	6 (30%)	0	0	2.05 (±0.76)	0.732
	7th year	7 (36.8%)	9 (47.4%)	1 (5.3%)	1 (5.3%)	1 (5.3%)	1.95 (±1.08)	
**B7.** I feel confident to assess an impacted mandibular third molar with respect to guidelines and recognise the need for surgical removal	6th year	5 (25%)	14 (70%)	0	1 (5%)	0	1.85 (±0.67)	0.015
	7th year	12 (63.2%)	7 (36.8%)	0	0	0	1.37 (±0.50)	
**B8.** I feel confident that I can recognise the clinical features of potentially malignant and malignant lesions of the oral cavity	6th year	1 (5%)	11 (55%)	6 (30%)	2 (10%)	0	2.45 (±0.76)	0.340
	7th year	1 (5.3%)	15 (78.9%)	2 (10.5%)	0	1 (5.3%)	2.21 (±0.79)	
**B9.** I feel confident that I can write an appropriate referral letter to a specialist in an appropriate time frame dependent on the clinical problem	6th year	0	9 (45%)	7 (35%)	4 (20%)	0	2.75 (±0.79)	0.086
	7th year	3 (15.8%)	10 (52.6%)	5 (26.3%)	0	1 (5.3%)	2.26 (±0.93)	
**B10**. I feel competent to differentiate between pain of odontogenic and non-odontogenic origin	6th year	1 (5%)	14 (70%)	5 (25%)	0	0	2.20 (±0.52)	0.971
	7th year	7 (36.8%)	4 (21.1%)	6 (31.6%)	1 (5.3%)	1 (5.3%)	2.21 (±1.18)	
**D1.** I believe my teaching in anatomy has been appropriate for my clinical needs in oral surgery	6th year	5 (25%)	10 (50%)	5 (25%)	0	0	2.00 (±0.73)	0.696
	7th year	7 (36.8%)	9 (47.4%)	1 (5.3%)	2 (10.5%)	0	1.89 (±0.94)	
**D2.** I am more confident about undertaking oral surgery because of my knowledge and understanding of head and neck anatomy	6th year	1 (5%)	10 (50%)	7 (35%)	2 (10%)	0	2.50 (±0.76)	0.777
	7th year	2 (10.5%)	10 (52.6%)	5 (26.3%)	1 (5.3%)	1 (5.3%)	2.42 (±0.96)	
**D3.** The only anatomical knowledge needed for oral surgery is that of jaw and tooth morphology	6th year	0	5 (25%)	0	5 (25%)	10 (50%)	4.00 (±1.26)	0.887
	7th year	1 (5.3%)	1 (5.3%)	0	11 (57.9%)	6 (31.6%)	4.05 (±1.03)	
**E1.** Oral surgery is an enjoyable and rewarding discipline	6th year	8 (40%)	4 (20%)	3 (15%)	5 (25%)	0	2.25 (±1.25)	0.477
	7th year	4 (21.1%)	13 (68.4%)	1 (5.3%)	0	1 (5.3%)	2.00 (±0.88)	

The majority of students (74.4%) expressed that they viewed oral surgery as an enjoyable and rewarding discipline. The majority of students had some experience at an off-campus dentistry centre (59%). However, less than half (41%) had carried out simple extractions at this off-campus site, and only two students (5.1%) had performed a surgical extraction off-campus (Table 3). In terms of experience in teeth extraction, 78.9% of seventh year students reported extracting more than 25 teeth to date while only 35% of sixth year students reported having had a similar amount of experience (Table 1).

**Table 3:** Responses to questions regarding off-campus learning

Question	Yes			No		
	6th year	7th year	Total	6th year	7th year	Total
**C1.** Where you involved in any rotation outside the faculty of dentistry dental centre (off-campus) scheme?	10 (50%)	13 (68.4%)	23 (59.0%)	10 (50%)	6 (31.6%)	16 (41.0%)
**C2.** Did you carry out any simple extractions outside the faculty of dentistry dental centre (off-campus)?	7 (35%)	9 (47.4%)	16 (41.0%)	13 (65%)	10 (52.6%)	23 (59.0%)
**C3.** Did you carry out any surgical extractions outside the faculty of dentistry dental centre (off-campus)?	2 (10%)	0	2 (5.1%)	18 (90%)	19 (100%)	37 (94.9%)

Statistically significant positive correlations were found between confidence in almost all aspects of surgical and forceps exodontia (Table 4). Only the correlation between confidence in extracting a single tooth and confidence in managing a failed extraction necessitating bone removal did not reach statistical significance but was borderline significant (*p* = 0.075).

**Table 4:** Spearman’s rho correlation coefficients (r) for questions in section B (forceps and surgical extractions)

Questions	B2	B3	B4a	B4b	B4c	B4d
**B2.** I feel confident that I could extract an upper single-rooted tooth with an intact crown, in an otherwise intact dentition	1.000	–	–	–	–	–
**B3.** I feel confident that I could remove visible retained roots of an upper left first molar with elevators or forceps	0.800**	1.000	–	–	–	–
**B4a.** I feel confident to assess and perform the surgical management of a failed extraction (e.g. a lower second molar) necessitating the raising of a mucoperiosteal flap	0.334*	0.322*	1.000	–	–	–
**B4b.** I feel confident to assess and perform the surgical management of a failed extraction (e.g. a lower second molar) necessitating Bone removal	0.288	0.326*	0.770**	1.000	–	–
**B4c.** I feel confident to assess and perform the surgical management of a failed extraction (e.g. a lower second molar) necessitating sectioning the tooth to facilitate elevation of the roots	0.361*	0.428**	0.786**	0.788**	1.000	–
**B4d.** I feel confident to assess and perform the surgical management of a failed extraction (e.g. a lower second molar) necessitating Wound closure using appropriate suture materials	0.615**	0.623**	0.683**	0.658**	0.721**	1.000

**P < 0.01, *P < 0.05 using a two-tailed test

The belief that anatomy teaching was appropriate (D1) correlated well with confidence in most aspects of surgical and forceps exodontia (Table 5). When questioned about confidence in undertaking oral surgery because of their knowledge and understanding of anatomy (D2), the only significant correlation was with confidence in managing a failed extraction necessitating the raising of a mucoperiosteal flap (B4a). This is because few students responded as “strongly agree” to this question. Furthermore, question D3 did not correlate with confidence in OMFS as students appeared to differ in their interpretation of this question which asked if knowledge of jaw and tooth morphology was the “only” anatomical knowledge needed for oral surgery. Table 5 also showed that confidence in undertaking OMFS was significantly correlated with the number of teeth students have extracted to date for all questions except B1 (The teaching that I have received in oral surgery has given me sufficient knowledge to undertake independent practice). This may have been due to a reluctance of students to strongly agree with this question.

**Table 5:** Spearman’s rho correlation coefficients (r) for questions in section B (forceps and surgical exodontia) and section D (anatomy teaching)

Questions	D1. I believe my teaching in anatomy has been appropriate for my clinical needs in oral surgery	D2. I am more confident about undertaking oral surgery because of my knowledge and understanding of head and neck anatomy	D3. The only anatomical knowledge needed for oral surgery is that of jaw and tooth morphology	F1. Total number of teeth extracted to date
**B1.** The teaching that I have received in oral surgery has given me sufficient knowledge to undertake independent practice	0.372*	0.241	0.127	−0.191
**B2.** I feel confident that I could extract an upper single-rooted tooth with an intact crown, in an otherwise intact dentition	0.258	−0.136	−0.209	− 0.418**
**B3.** I feel confident that I could remove visible retained roots of an upper left first molar with elevators or forceps	0.168	−0.185	−0.019	− 0.484**
**B4a.** I feel confident to assess and perform the surgical management of a failed extraction (e.g. a lower second molar) necessitating the raising of a mucoperiosteal flap	0.584**	0.361*	0.005	−0.382*
**B4b.** I feel confident to assess and perform the surgical management of a failed extraction (e.g. a lower second molar) necessitating Bone removal	0.468**	0.199	−0.293	−0.502**
**B4c.** I feel confident to assess and perform the surgical management of a failed extraction (e.g. a lower second molar) necessitating sectioning the tooth to facilitate elevation of the roots	0.484**	0.130	−0.135	−0.425**
**B4d.** I feel confident to assess and perform the surgical management of a failed extraction (e.g. a lower second molar) necessitating Wound closure using appropriate suture materials	0.320*	−0.020	−0.140	− 0.458**

**P < 0.01, *P < 0.05 using a two-tailed test

## Discussion

In recent years, there has been an increase in the number of dental schools around the world with differing standards of undergraduate teaching, the number of years in training, and the numbers of qualified teaching faculty members [4, 7, 8]. This makes it difficult to compare graduates from these dental schools. A study by Lee et al. demonstrated that undergraduate grades and scores on the standardised dental admission test are poor predictors of performance in examinations used for residency admission in OMFS [11]. A major part of any dentistry curriculum relies on helping students acquire predefined clinical competencies and technical skills, and many consider the mastery of these technical skills to be of the highest importance in clinical practice [3, 6]. Thus, clinical competency and self-confidence in one’s abilities are the primary objectives of any dental school curriculum [3]. Self-assessment of students’ knowledge and confidence to complete clinical skills have previously been used in the field of dentistry and oral surgery [1–4, 7–10, 12–15].

Given that the department of OMFS at Kuwait University is a relatively new one (established in 2002), feedback from students is of paramount importance to improve the quality of teaching delivered in the department. The high response rate seen in this study reflects the interest of students to voluntarily assess their undergraduate OMFS teaching in preparation for their entry in the dentistry practice. The majority of the dental students were females, which indicates the overall interest of the female students in Kuwait to embark on a career in the field of dentistry. Even though the class sizes are small, the responses of the students were regarded as promising as they feel confident that they have enough knowledge to undertake independent practice (61%). This is even greater (78.9%) when only those in their final year of university are considered.

Overall, when assessing readiness to undertake private practice and perform extractions with forceps and minor oral surgery procedures, confidence scores were favourable and similar to previous studies utilising the same survey instrument [3, 4, 7–10].

Both the sixth and seventh year students reported confidence in extracting an upper single-rooted tooth with an intact crown (94.7 and 90% respectively). In addition, both groups were also confident that they could remove visibly retained roots of an upper left first molar with elevators or forceps (84.2 and 65% respectively). This finding was different from a study by Burdurlu et al., in which the older class reported being more confident than those in lower year groups [8]. This may be due to the fact that dental students at Kuwait University undergo a more extensive teaching curriculum with a longer study period, where dentistry studies take 7 years (with the last two and half years as clinical years), compared to the study by Burdurlu et al., where the study programme was of a five-year duration. Nevertheless, the responses of the sixth and seventh year students were statistically different when reporting on their level of confidence for performing surgical procedures, ranging from raising of a mucoperiosteal flap, sectioning of teeth, bone removal, wound closure, and suturing (Table 2). This was in line with other studies which reported relatively lower self-confidence in conducting surgical extractions [4, 8, 9, 14].

Responses about the level of confidence in diagnosing and managing acute pericoronitis, assessing impacted third molars, or managing haemorrhage from a socket were more favourable than recognising benign and malignant conditions, differentiating pain origins, or writing detailed referral letters to other specialists (Table 2). Similar findings were also demonstrated in the studies by Cabbar, Burdurlu, and Macluskey [7–10]. One explanation to why most students score relatively low in confidence in conducting surgical extractions is that they are considered the most invasive procedure that students are exposed to during their dental school training, and even if they are clinically competent as dentists, they may feel intimidated by it [9, 16].

When assessing anatomical knowledge, the responses from the students indicate that their teaching was sufficient to prepare them for OMFS clinical scenarios, and the responses were not significantly different between sixth and seventh year students. This may be because the students receive extensive didactic OMFS teaching in their clinical years with a strong emphasis on head and neck anatomy. The importance of instilling constant anatomical knowledge during dental education to help with consolidation and retention of the clinical knowledge was advocated by Thomas et al. [8, 17]. The fact that both classes disagreed unequivocally to the item that only anatomical knowledge was needed for oral surgery is that jaw and tooth morphology show a maturation of their understanding that general anatomy knowledge is of paramount importance when treating patients or performing oral surgical procedures.

Only 59% of students reported that they had the opportunity to gain experience in dental centres off-campus. This gave students the chance to perform more simple extractions with some being allowed to perform surgical extractions. The role of off-campus learning needs to be emphasised in our teaching to increase the exposure of the students to the more complex procedures that are not heavily emphasised in the dental school’s clinic.

In our study we found that students in both junior and senior clinical years have sufficient levels of confidence to perform extractions by the use of forceps, and a good higher level of confidence when diagnosing conditions commonly seen in oral surgery practice, such as managing acute pericoronitis, and haemorrhage from a socket, assessing impacted teeth, and recognising the clinical features of potentially malignant and malignant lesions of the oral cavity. However, both year groups showed a lower level of self-confidence in performing more invasive procedures such as the raising of a flap, sectioning of teeth and bone removal, and wound closure with suturing. This prompts us to put more emphasis on hands-on training sessions, utilising phantom heads in oral surgical education, assisting in major surgical procedures, and utilising novel models to conduct these surgical procedures which are considered to be essential for dentists wanting to practice the whole spectrum of general dentistry in clinical practice.

A limitation encountered in this study was that dental students have different performance and academic calibre, and this were not adequately assessed by our methodology. Given the nature of the dental curriculum, students were evaluated mostly through institution-based didactic examinations and through grading supervised competency in performing limited numbers of procedures. The true calibre and performance level of the students could thus be under-evaluated. Further detailed and standardised didactic and clinical assessment tools need to be introduced to better understand and evaluate the students’ performance in OMFS.

## Conclusions

Students felt confident undertaking independent practice and preforming exodontia using forceps and elevators, root removal, managing acute pericoronitis, managing haemorrhage from a socket, assessing impacted teeth, and recognising the clinical features of potentially malignant and malignant lesions of the oral cavity. They reported a lower level of confidence for performing surgical procedures, ranging from the raising of a mucoperiosteal flap, sectioning of teeth, bone removal, wound closure and suturing, to the writing of a detailed referral letter to specialists. This should prompt us to increase their clinical exposure to these procedures in the faculty clinic and off-campus rotations and refine our training schemes in the areas of surgical extractions and procedures related to minor oral surgery, such as raising a flap, bone removal, root section, and wound suturing. The ABAOMS survey instrument is a useful and thorough tool to assess the self-confidence of dental students according to their undergraduate oral and maxillofacial surgery teaching.

## Data Availability

The data used in this study is not publicly available, but data can be made available from the corresponding author on request.
